# Potential animal reservoirs (dogs and bats) of human visceral leishmaniasis due to *Leishmania infantum* in French Guiana

**DOI:** 10.1371/journal.pntd.0007456

**Published:** 2019-06-19

**Authors:** Hacène Medkour, Bernard Davoust, François Dulieu, Laurent Maurizi, Thierry Lamour, Jean-Lou Marié, Oleg Mediannikov

**Affiliations:** 1 Aix Marseille Univ, IRD, AP-HM, MEPHI, IHU Méditerranée-Infection, Marseille, France; 2 IHU Méditerranée Infection, Marseille, France; 3 Animal Epidemiology Working Group of the Military Health Service, Marseille, France; 4 24^e^ Veterinary Group, Suippes, France; 5 27^e^ Veterinary Group, Metz, France; 6 Army Health Service of French Guiana, Cayenne, France; 7 French Army Health Service, Paris, France; Institute of Tropical Medicine, BELGIUM

## Abstract

In French Guiana, cutaneous leishmaniasis is highly endemic, whereas no autochthonous case of visceral leishmaniasis have been reported so far. However, due to its proximity to Brazil which is highly endemic for visceral leishmaniasis, and the high transboundary population flow, an epidemiological challenge could arise at any time. As an overseas department and region and the largest outermost region of the European Union, epidemiological surveillance of visceral leishmaniasis is of great importance. Our study aimed to investigate the presence of *Leishmania* spp. in domestic (dogs) and sylvatic (bats) animals from French Guiana. Over the 2008–2018 period, samples from 349 animals were collected. They included blood from 179 autochthonous dogs and 59 bats, spleen samples from 33 bats and, blood from 78 military working dogs (MWD) collected before their departure from continental France and at the end of their four-month stay in French Guiana. Samples were screened using real-time polymerase chain reaction (qPCR) assays targeting *Leishmania* DNA followed by sequencing of 18S rRNA, kDNA and ITS2 genes. *L*. *infantum* was detected in 2.3% (8/349) of animals with 1.7% (3/179) of autochthonous dogs, 5.1% (4/78) of MWD returning from French Guiana, whereas they were negative before their departure. One of them dates back to 2012. All these dogs were positive for serological tests. In addition, *L*. *infantum* DNA was detectable in one bat spleen sample, belonging to *Carollia perspicillata* species. We report here for the first time an infection with *L*. *infantum* in dogs and bat from French Guiana. Our results suggest the existence of potential reservoir and transmission cycle for visceral leishmaniasis, at least since 2012, which was unknown in this territory until now. Further studies are needed to determine how these animals were infected and which vectors are involved in the transmission in this area.

## Author summary

Leishmaniasis is endemic in French Guiana under its cutaneous form where *Leishmania guyanensis* is the principle parasite species. Visceral leishmaniasis is much more severe, although well known in neighbouring countries (Brazil, Suriname, Venezuela), it has not been known in French Guiana until now. Our study presents the result of a ten-year surveillance of *Leishmania* spp. infections in dogs and bats from French Guiana. We analyzed 92 bats from French Guiana 179 local dogs and 78 additional French military working dogs (MWD), which spent a short stay in this territory. Globally, we found 2.3% (8/343) of *Leishmania* infected animals. *L*. *infantum* infection was detected in 1.7% (3/179) of autochthonous dogs and 5.1% (4/78) of MWD. One of them dates back to 2012, the others were in 2016 and 2018. Low infection rate was detected in bats, only one specimen among 92 being infected (1.1%) and belonging to *Carollia perspicillata* species. We conclude that *L*. *infantum* has been circulating in French Guiana since at least 2012. Dogs and bats could therefore be among the potential reservoirs. Further investigations on potential additional reservoirs among domestic and wild animals, as well as identification of vectors, are required.

## Introduction

Leishmaniasis are among of the ‘‘most neglected diseases”[1] from the group of vector-borne diseases. Leishmaniasis are caused by parasites belonging to the genus *Leishmania* (Trypanosomatida: *Trypanosomatidae*) with a worldwide distribution in large areas of the tropics, subtropics and Mediterranean basin, involving more than 98 countries [2]. The burden of leishmaniasis increased over the last decades, making them among tropical infections, the 2^nd^ and 4^th^ most common cause of death and disease, respectively [3]. Worldwide, the population of 350 million is at risk with an annual incidence of 0.7–1.2 million cases of cutaneous leishmaniasis (CL) and 0.2–0.4 million cases of visceral leishmaniasis (VL) [4]. VL is often caused by *L*. *donovani* and *L*. *infantum* (syn *L*. *chagasi*) and involves various mammals such as humans and dogs [3, 5]. The parasite infects internal organs, such as spleen, liver and bone marrow. Dogs are the main reservoir and hosts of *L*. *infantum* causing canine leishmaniosis (CanL), a severe systemic disease reported in more than 70 countries and common in the Mediterranean region and in South America [5, 6]. It is estimated that 2.5 million dogs are infected in the Mediterranean basin only [7]. Some VL cases, caused by *L*. *tropica* or *L*. *amazonensis*, have also been reported [8]. The main vectors belong to *Euphlebotomus*, *Larroussius* and *Synphlebotomus* sandfly subgenera in the Old World [9], and *Lutzomyia* subgenus in the New World [10, 11]. Natural cycle of CL involves various vertebrate hosts (wild rodents and humans) and different sandfly species as vectors for its spread. Its etiological agents include *L*. *tropica*, *L*. *major*, and *L*. *aethiopica* transmitted especially by *Phlebotomus* and *Paraphlebotomus* sandflies in the Old World [12]. However, New World CL is caused by *L*. *mexicana* complex (*L*. *mexicana*, *L*. *amazonensis*, etc.) or the subgenus *Viannia* (*L*. *braziliensis*, *L*. *guyanensis*, etc.) and occurs especially in tropical and subtropical areas of Mexico and Central and South America. The main vectors are classified in the subgenera *Nyssomyia*, *Psychodopygus*, *Lutzomyia* s.str., and *Verrucarum* [11].

French Guiana is situated in the northern part of South America between Brazil and Suriname and extends on 84 000 km^2^. Its climate is equatorial (hot and humid) and the Amazonian forest covers 90% of its territory. Like the entire Amazon region, French Guiana hosts many wild and domestic animals that act as reservoirs for pathogens and the emergence of infectious diseases, amongst them, various zoonotic diseases. CL has been known here since 1943 [13] and cases have been reported regularly since then. The annual incidence rate was estimated between 15 and 20 new cases per 10,000 inhabitants between 1979 and 2012 [14, 15]. *L*. *guyanensis* is undoubtedly the most common species, and other species implicated in CL has increased over the years: *L*. *braziliensis*, *L*. *amazonensis*, *L*. *lainsoni*, and *L*. *naiffi* [16, 17]. Human and canine VL are widespread and are endemic in many areas of Latin America [18]. Although the principal foci of visceral leishmaniasis are located in the drier, poorly forested areas, a small number of human and canine infections have been recorded in the densely forested Amazon region, such as in the State of Para. In Roraima, a particularly large focus also extends to Venezuela and Guyana. In Venezuela, it occurs sporadically in almost every state of the country with a low endemicity. Till now, VL has been reported several times in Suriname [19] but never in French Guiana [20]. In 2006, two imported cases of canine VL in Cayenne, the main city of French Guiana, were reported; one infected dog was most probably imported from France. A second dog was then infected with *Leishmania infantum* in French Guiana [21].

Globally, *Leishmania* spp. natural infections have been repeatedly reported in domestic, peridomestic and wild animals, dogs and rodents being the most investigated animals and considered as reservoirs [22]. However, recent investigations of *Leishmania* in animals have drawn attention to other possible sylvatic reservoir hosts in endemic leishmaniasis foci such as hares [23], marsupials [24], wild canids such as bush dog (*Speothos venaticus*) [25, 26], bats, primates and numerous other mammals [24, 27, 28]

The present study represents a ten-year surveillance (from 2008 to 2018) of the *Leishmania* circulation in dogs and bats in French Guiana using molecular and serological techniques.

## Materials and methods

### Ethics statement

All dogs sampled in this study were examined with the assistance and acceptance of their owners. Blood samples were collected by veterinarians according to the good practices of veterinary medicine. Article R.214-88 of the French Rural Code and Sea Fishing (Decree No. 2013–118 of 1 February 2013 on the protection of animals used for scientific purposes) excludes these acts from the scope of applications for authorization granted by the Minister responsible for research. The protocol for capturing and sampling bat specimens (N°1688) was approved by the Animal Ethics Committee of Marseille (C2EA14) and by the French authorities.

### Animals

**Fig 1:**
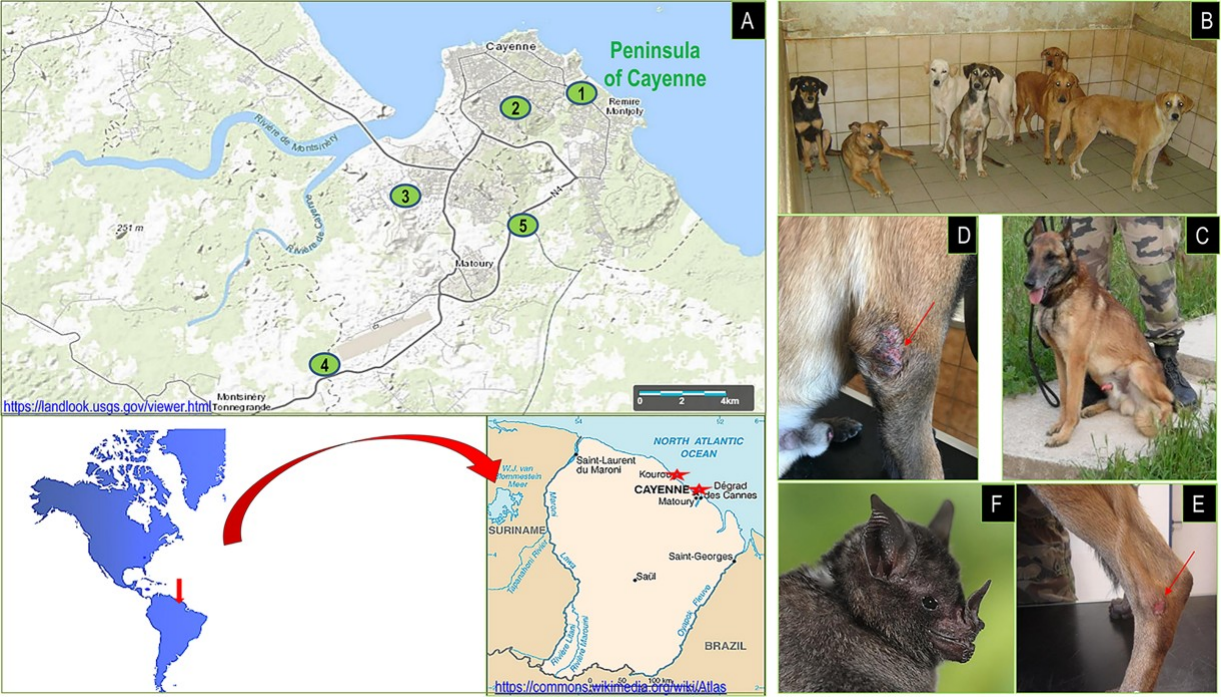
Study area and animal locations. **A.** Locations of *L*. *infantum*-infected animals, circles 1, 2 and 3: tree autochthonous dogs, circle 4: 4 MWD, circle 5: Bat **(**copyright map: https://commons.wikimedia.org/wiki/Atlas_of_French_Guiana; https://landlook.usgs.gov/viewer.html ; **B.C.** French Guiana autochtonus dogs and MWD (copyright pictures: B. Davoust). **D.E.** *L*. *infantum* skin lesions on MWD2 and MWD1 (copyright pictures: F Dulieu, L Maurizi). **F.** Bat *Carollia perspicillata* (copyright picture: JM Bompar).

This study was conducted between 2008 and 2018. One hundred dogs were studied in Cayenne (4° 56′ 4.6″ N, 52° 19′ 49.19″ W), the capital of Guiana with 55,000 residents. Some 60 km northwest of Cayenne, in Kourou, we collected samples from 79 dogs (5° 9′ 34.92″ N, 52° 39′ 1.08″ W) (Fig 1). Dogs came from dog shelters from both cities and from private dog owners in Cayenne, who gave their consent. Overall, blood samples were taken from 179 adult dogs, all apparently healthy, including 107 females and 72 males, of which 26 were sampled in 2008, 55 in 2014 and 98 in 2016. In addition, between 2012 and 2018 period, pairs of blood samples have been collected from 78 military working dogs (MWD) before their departure from metropolitan France and after their return from a four-month stay in French Guiana. All of them were males and the majority was Berger Belge Malinois. Most of them stayed in Cayenne but some of them were involved in missions in the deep forest. We sampled 16 dogs in 2012, 26 in 2016, 20 in 2017 and 16 in 2018. All MWD were apparently healthy, except two of them: one (MWD1) had an ulcer on the hock and one (MWD2) had crusts at the elbows and skin lesions, after their return from French Guiana in 2018. For this last (MWD2), a skin swab and a bone marrow aspiration have been implemented for further *Leishmania* DNA detection. For dogs, blood collection was performed by cephalic venipuncture using EDTA tubes; all samples were stored at + 4°C until being transported to our laboratory. Ethical aspects relating to dog sampling was made in accordance to the French law.

On the other hand, 92 apparently healthy bats were captured as follows: in 2013, blood samples were collected from 59 bats (32 males and 27 females) in five areas near the coast of French Guiana: Cayenne (04° 54’ 65” N–52° 18’ 54” W),Kourou (05° 13’ 96” N–52° 45’ 19° W), Saint-Jean-du-Maroni (05° 23’ 95” N–54° 04’ 72” W), Crique Malmanoury (05° 09’ 61” N–52° 53’ 59” W), and Regina (04° 17’ 93” N–52° 11’ 88” W). In 2014, spleen samples have been recovered aseptically from 33 bats (18 males and 15 females) from Cayenne (04° 54’ 65” N–52° 18’ 54” W). Bats were trapped using hand nets, mist nets or by hand. Catches occurred in residential or unoccupied buildings, in culverts beneath roads, under bridges, or in caves. The species, sex, reproductive status and morphological measurements of the bats were recorded. All samples were stored at + 4°C until transportation to the lab and were subsequently deep freezed until analysis.

### DNA extraction

We extracted DNA from 200 μL blood of dogs and bats after digestion with 15μL of proteinase K (20mg/mL) at +56°C overnight. DNA from bat spleens was purified from approximately 25 mg of starting material mixed in 200 μL of buffer, 20 μL proteinase K and ≈20 mg of glass powder, followed by 30 sec lyse in FastPrep-24 device (Sample Preparation system) from MP Biomedicals, USA and incubation at +56°C overnight. DNA was extracted using a commercial DNA extraction kit (QIAamp DNA Mini Kit, [Qiagen, Courtaboeuf, France]) on BIOROBOT EZ1 (Qiagen, Qiagen, Courtaboeuf, France) according to the manufacturer’s instructions. It was then eluted in 200 μL of distilled water and stored at -20°C under sterile conditions to farther use for PCR-based amplification.

### Primers and probes

*Leishmania* DNA detection and species identification were performed by PCR/sequencing. In order to detect the *Leishmania* DNA, we performed the qPCR-based screening of samples with qPCR assay using primers and probe targeting a portion of small subunit of the 18S ribosomal gene (18S rRNA) to detect the presence of the genus *Leishmania* [2]. Positive samples were confirmed by a conventional PCR (PCR) targeting 550 bp fragment length from the same gene followed by sequencing, allowing the identification of *Leishmania* at the complex level (Medkour et al., submitted). Samples were also analyzed by qPCR using primers and probe for detection and quantification of *L*. *infantum* DNA with high sensitivity, targeting a conserved region of the kinetoplast minicircle DNA (kDNA) (several 1000’s-fold repeated sequence), as described previously [29, 30, 31]. Positive samples were confirmed by PCRs/sequencing using primers RV1/RV2 targeting 140 pb of kDNA gene, primers LGITSF2/LGITSR2, targeting of 370 to 450 bp fragment of internal transcribed spacer 2 gene (ITS2); to identify *Leishmania* species. Primers and probes, their conditions and sources are listed in Table 1.

**Table 1:** Sequences of primers set used for *Leishmania* detection and species identification.

Targeted microorganisms	PCR	Target gene	Name	Primers (5’-3’) and probe	Tm	References
***Leishmania* spp.**	qPCR	18S rRNA	Leish. F	GGTTTAGTGCGTCCGGTG	60°C	Medkour et al. submitted
			Leish. R	CGGCCCATAAGATCC CCAA		
			Leish. P*	FAM-CGGCCGTAACGCCTTTTCAACTCA -TAMRA		
***L*. *infantum***	qPCR	kDNA	RV1	CTTTTCTGGTCCTCCGGGTAGG	60°C	[31]
			RV2	CCACCCGGCCCTATTTTACACCAA		
			Probe. Leish*	FAM-TTTTCGCAGAACGCCCCTACCCGC-TAMRA		
***Leishmania* spp.**	PCR	18S rRNA	Leish. F1	CTGTGACTAAAGAAGCGTGAC	52°C	Medkour et al. submitted
			Leish. R1	AGGCCGAATAGAAAAGATACGT		
		kDNA	RV1	CTTTTCTGGTCCTCCGGGTAGG	59°C	[32]
			RV2	CCACCCGGCCCTATTTTACACCAA		
		ITS 2	LGITSF2	GCATGCCATATTCTCAGTGTC	60°C	[33]
			LGITSR2	GGCCAACGCGAAGTTGAATTC		

Abbreviations

**Tm**: Annealing temperature; *: Probe

### Polymerase chain reaction amplification, sequencing and phylogeny

For all DNA samples, qPCR assays based on 18S rRNA and kDNA were prepared in a final volume of 20 μL with 10 μL of Eurogentec Master Mix Roche (Eurogentec, Liège, Belgium), 3 μL of distilled water DNAse and RNAse free, 0.5 mM of each primer and 0.5 mM of the FAM- labeled probes (Table 1), 0.5 μL UDG and 5 μl of the DNA template. The amplification was performed in a CFX96 Real-Time system (BioRad Laboratories, Foster City, CA, USA) using the following thermal profile: one incubation step at 50°C for two minutes and an initial denaturation step at 95°C for three minutes, followed by 40 cycles of denaturation at 95°C for 15 seconds and annealing extension at 60°C for 30 seconds. DNA from cultured *L*. *infantum* and *L*. *donovani* were included as positive controls and master mixtures as a negative control for each assay. Results were considered positive when the cycle threshold (Ct) was lower than 38 Ct and 35 Ct for 18S rRNA- and kDNA-based qPCRs, respectively.

For the kDNA qPCR assay, the standard curve of amplification was 8 folds-serial dilution of plasmid of 10^8^ copy of DNA/mL from the kDNA region, equivalent of 10000 parasites/mL; 5 μl of serial dilutions ranging from 10000 to 0.001 parasites/mL, was introduced into reaction tubes. For blood, results were expressed as the number of *Leishmania* parasites present in 1 ml of blood, taking in account the volume (200 μl of blood) and the elution (200 μl) introduced during the extraction process. For bone marrow, skin lesion and spleen, number of *Leishmania* parasites present in 1 g of tissue taking in account the quantity (20 mg environ) and the elution (200 μl) volume introduced during the extraction.

Samples positive by qPCR were amplified in conventional PCR assays mentioned above (Table 1). PCR reactions contained 5 μl of DNA template, 25 μl AmpliTaq Gold 360 Master Mix from Applied Biosystems (Thermo Fisher Scientific), 1 μl of each primer and water to create a final reaction mixture volume of 50 μl. Amplifications were performed in a Peltier PTC-200 model thermal cycler (MJ Research Inc., Watertown, MA, USA) under the thermal cycling conditions: one incubation step at 95°C for 15 minutes, 40 cycles of one minute at 95°C, 30 seconds annealing at the corresponding temperature (Table 1) and one minute at 72°C followed by a final extension for five minutes at 72°C. Visualization was performed in electrophoresis on 2% agarose gels. Master mixture and DNA from cultured *L*. *infantum* and *L*. *donovani* were included as negative and positive controls, respectively, in each assay. Purification of PCR products was performed using NucleoFast 96 PCR plates (Macherey-Nagel EURL, Hoerdt, France) according to the manufacturer's instructions. The amplicons were sequenced using the Big Dye Terminator Cycle Sequencing Kit (Perkin Elmer Applied Biosystems, Foster City, CA) with an ABI automated sequencer (Applied Biosystems). The obtained electropherograms were assembled and edited using ChromasPro software (ChromasPro 1.7, Technelysium Pty Ltd., Tewantin, Australia) and compared with those available in the GenBank database by NCBI BLAST (https://blast.ncbi.nlm.nih.gov/Blast.cgi). All sequences were deposed in the GenBank database. Molecular Phylogenetic analysis was performed with MEGA version 7 software [34].

### Leishmania antibodies detection

Leishmania antibodies were also tested on the PCR positive blood samples by Rapid immuno-migration (RIM) using Witness Leishmania commercial test (Zoetis, Lyon, France), which uses an antigen from *L*. *infantum* to quickly identify antibodies in blood, sera or plasma from *Leishmania*-infected animals. When available, indirect immunofluorescence antibody test (IFAT) was performed for some MWD and Leishmania antibodies were then quantified [35].

### Statistical analysis

Data were collected and described in XLSTAT version 2018.7.

## Results

Molecular assays showed a prevalence of 1.7% (3/179) for *L*. *infantum* infection in autochthonous dogs from Guiana, with: 0% (0/26) in 2008, 0% (0/55) in 2014 and 3.1% (3/98) in 2016 (Table 2). These three PCR positive samples were also found positive by serology using the RIM test (Table 3). In addition, four MWD (4/78, 5.1%) were diagnosed infected upon their return to France after a four-month stay in Guiana. One MWD was diagnosed in 2012 and another in 2016. In 2018, 2 other MWDs from the group of 16 dogs (12.5%) were infected during their stay in French Guiana (Table 2). All the MWDs were negative for both PCR and serology before their stay in French Guiana. The two infected MWDs in 2018 showed presence of *L*. *infantum* antibodies by RIM and by IFAT at dilution 1/200 for one (MWD1) and 1/3600 for the other (MWD2). These two dogs presented signs of CanL (Fig 1), so, among the 7 positive dogs (PCR and/or serology), two were symptomatic and 5 asymptomatic. Symptomatic dogs were examined and treated by military veterinarians using the classical treatment (Glucantime + Allopurinol). Overall, the mean parasite load in dog’s blood was 10921 (min: 3.7; max: 42390) parasites/mL. We detected 33,940 parasites/mL blood on the MWD1. In the sick MWD2, we detected: 4.210^4^ parasites/mL of blood, 1.310^6^ and 6.6 10^6^ parasites/g from skin lesion and bone marrow, respectively (Table 3).

**Table 2:** Molecular results for surveyed animals.

Animals Years			No. of dogs	q PCR, No. Pos (%)		Conventional PCR, No. Pos		
				18S rRNA	kDNA	18S rRNA	kDNA	ITS2
**Autochthonous dogs**	2008	-	26	0 (0)	0 (0)	-	-	-
	2014	-	55	0 (0)	0 (0)	-	-	-
	2016	-	98	3 (3.1)	3 (3.1)	3	3	3
	Total		**179**	3 (1.7)	3 (1.7)	3	3	3
**Military working dogs**	2012	Departure	16	0 (0)	0 (0)	-	-	-
		Return	16	1 (6.2)	1 (6.2)	1	1	1
	2016	Departure	26	0 (0)	0 (0)	-	-	-
		Return	26	0 (0)	1 (3.8)	0	0	0
	2017	Departure	20	0 (0)	0 (0)	-	-	-
		Return	20	0 (0)	0 (0)	-	-	-
	2018	Departure	16	0 (0)	0 (0)	-	-	-
		Return	16	2 (12.5)	2 (12.5)	2	2	2
	Total departure		78	0 (0)	0 (0)	-	-	-
	Total return		78	3 (3.8)	4 (5.1)	3	3	3
**Total dogs**			**257**	6 (2.3)	7 (2.7)	6	6	6
**Bats**	2013	Blood	59	0 (0)	0 (0)	-	-	-
	2014	Spleen	33	1 (3)	1 (3)	1	1	1
**Total bats**			**92**	1 (1.1)	1 (1.1)	1	1	1
**Total animals**			**349**	7 (2)	8 (2.3)	7	7	7

Conventional PCR had been performed for positive samples by one or the two qPCR assays. **No.**: Number; **Pos:** positive

**Table 3:** Parasite load and serological test results for qPCR-positive samples.

Sample	Animal	Ct PCR (kDNA)	No. parasite/mL or g	Witness Leishmania	IFAT
**Blood**	CMT 21	25.6	11.7	+	ND
	CMT 80	29	14.7	+	ND
	CMT 95	22.6	76.1	+	ND
	MWD A	30.4	14.5	+	ND
	MWD B	34.6	3.7	+	ND
	MWD1	16.5	33,940	+	1/200
	MWD2	16.2	42,390	+	1/3,600
**Bone marrow**	MWD2	8	6,647,000	ND	ND
**Skin scratch**	MWD2	10.9	1,300,000	ND	ND
**Spleen**	BAT	26.8	55.6	ND	ND

**Abbreviations: Ct:** cycle threshold; **No. of parasite/mL or g:** Number of *Leishmania* parasites by mL of blood or g of other tissues (bone marrow, skin or spleen); **IFAT**: indirect immunofluorescence antibody test; **CMT**: autochthonous dog; **MWD**: military working dog; **ND:** not determined.

Blood samples were collected from 59 bats in 2013, according to the following species distribution: 10 *Eumops auripendulus*, 8 *Artibeus planirostris*, 22 *Noctilio albiventris*, 2 *Molossus barnesi*, 14 *Pteronotus parnellii*, 1 *Phyllostomus elongatus* and 2 *Carollia perspicillata*. No blood samples taken from these species were found positive. In addition, 33 spleen samples were collected from *Carollia perspicillata* in 2014 and *Leishmania* DNA was detected in one of them (3%, 1/33). The total prevalence of *L*. *infantum* infection in bats observed in this study was 1.1% (1/92) and the parasite load was 55.6 parasites/g in spleen. The infected bat, *Carollia perspicillata* (Fig 1), did not show any lesions and no apparent macroscopical lesions were found in its organs.

### Sequencing

**Fig 2:**
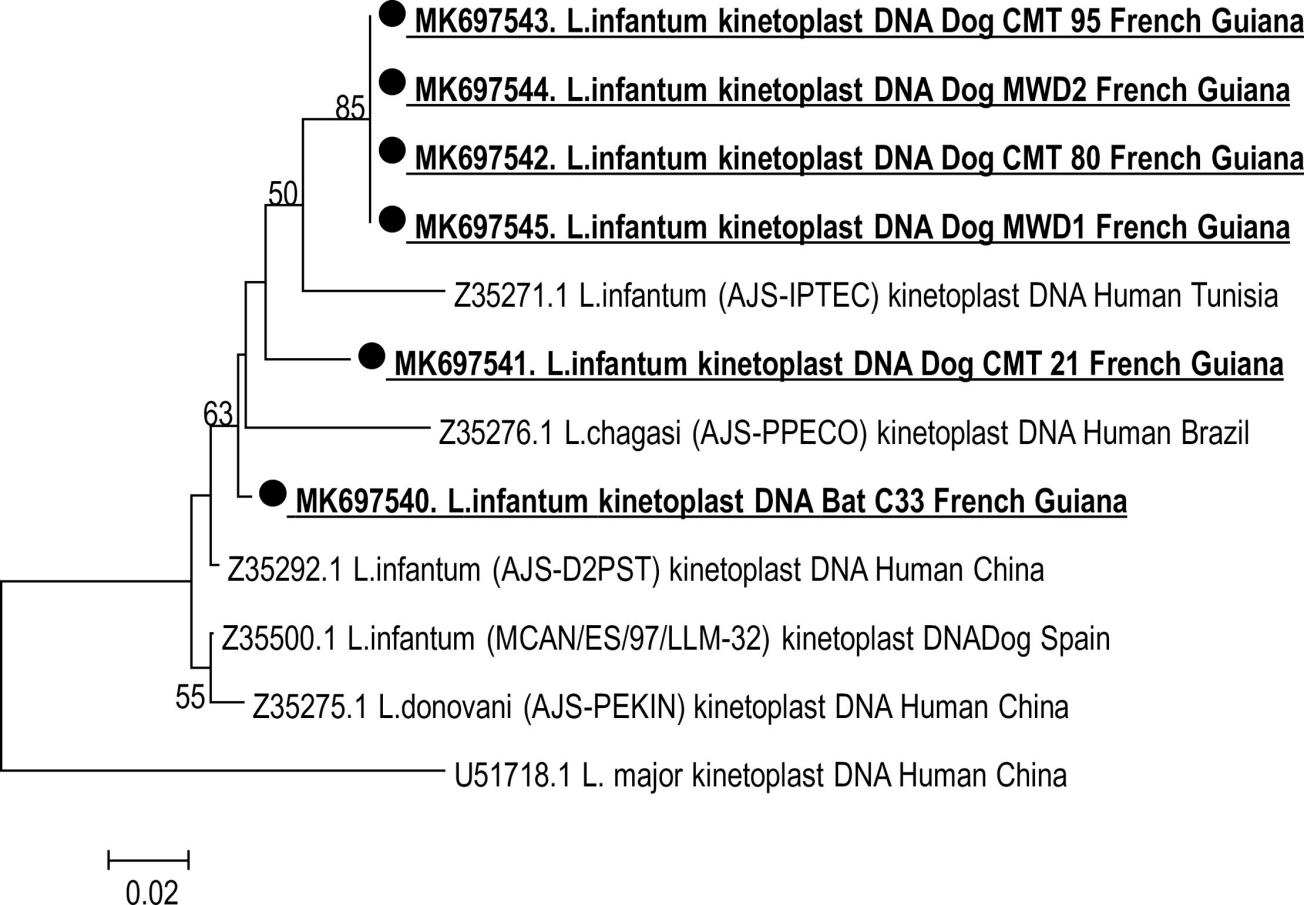
Phylogenetic tree constructed based on the sequences of Kinetoplast minicircle gene for isolates in this study and other isolates of the *Leishmania* species from GenBank database. Neighbor-joining tree was constructed from Kinetoplast gene using MEGA 7.0 software. The Kimura-2-parameter method was used. Numbers above branches correspond to bootstrap values based on 1,000 replicates. Bootstrap low to 50 were removed. The analysis involved 12 nucleotide sequences. All positions containing gaps and missing data were eliminated. There were a total of 119 positions in the final dataset. Isolates were designated by their accession numbers in the beginning and their names.

**Fig 3:**
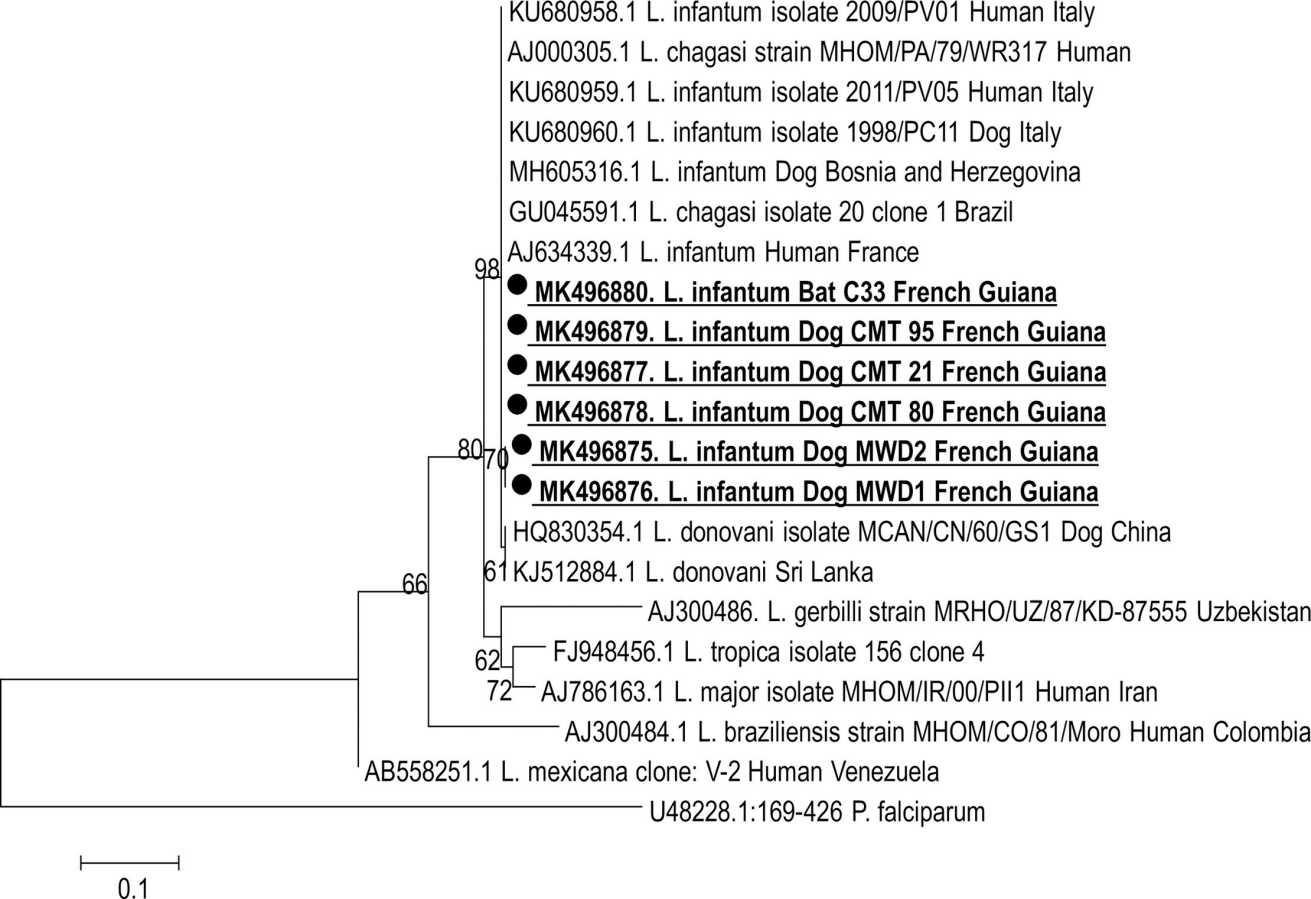
Maximum likelihood phylogenetic tree based on *Leishmania* ITS2 sequences showing the relationships of the obtained sequences in this study of and other isolates of the *Leishmania* species from GenBank database. The evolutionary history was inferred by using the Maximum Likelihood method based on the Tamura-Nei model. The tree is drawn to scale, with branch lengths measured in the number of substitutions per site. The analysis involved 21 nucleotide sequences. All positions containing gaps and missing data were eliminated. There were a total of 223 positions in the final dataset. Evolutionary analyses were conducted in MEGA 7.0 software.

Of the 7 (6 from dogs and one from bat) amplicons obtained for the 18S rRNA, kDNA and ITS2 genes, 6 (5 from dogs and one from bat) had been sequenced. Sequencing analysis of these genes concludes for *L*. *infantum* species. The 18S rRNA obtained sequences of 520 to 537-bp fragment length were almost identical and had 99–100% similarity with *L*. *donovani* complex available sequences in GenBank database such as: *L*. *donovani* (a.n. GQ332356) and many other *L*. *infantum/chagasi* sequences: *L*. *infantum* JPCM5 isolate (a.n. XR 001203206) and *L*. *chagasi* C29 isolate (a.n. KT762398) (Supplementary Fig 1). Four sequences of 140-bp of the kDNA gene obtained from two local dogs and two MWDs were identical and showed 97% similarity with *L*. *infantum* MCAN/ES/98/10445 clone LinGpja 8 detected on a Spanish dog (a.n. EU437406.1). Further amplicons of 120-bp of the kDNA gene from a local dog and a bat showed 92% identity with *L*. *chagasi* AJS-PPECO isolate (a.n. Z35276) detected in a human in Brazil and 97% identity with *L*. *infantum* AJS-IPTRS isolate (a.n. Z35274) from a human in Tunisia, respectively (Fig 2). For the ITS2 gene sequencing, two amplicons of 390 from a local dog and a bat and two others of 420 bp from two local dogs were closely identical to each other and with many *L*. *infantum/chagasi* ITS2 gene published sequences in GenBank database, i.e. *L*. *infantum* (a.n. AJ634339) detected in a human in France and *L*. *chagasi* isolate 20 clone 1 (a.n. 1GU045591) from Brazil. Two MWDs ITS2 amplicons of 360 bp were identical and exhibited 99% with *L*. *infantum/chagasi* cited above (Fig 3).

## Discussion

The Amazon rainforest, with luxurious vegetation and high rainfall (> 3 m / year) is an environment that promotes both multiplication of vectors and mammalian reservoir hosts. The demographic development of the population and the anthropisation of the environment facilitate the *Leishmania* cycle development. These features are found in almost all the Guianas. So far, two parasitic cycles of leishmaniasis have been described in French Guiana. *L*. *guyanensis* occurs in the canopy with the arboreal sand fly *Lutzomyia umbratilis* as a vector, the two-fingered sloth, *Choloepus didactylus*, as main reservoir and the opossum *Didelphis marsupialis* as secondary reservoir. The second cycle concerns especially *L*. *amazonensis*, evolving at the ground level with *Lutzomyia flaviscutellata* as vector and the spiny rat *Proechymis cuvieri* as reservoir [36]. In this study, and for the first time, we report the presence of *L*. *infantum* in dogs and bat in French Guiana, suggesting the existence of a potential new domestic and wild reservoir of VL in this area where no indigenous human cases have yet been reported.

Our ten-year longitudinal study (2008–2018) showed *L*. *infantum* infection in surveyed animals. In French Guiana, literature reports no case of *L*. *infantum* infection since the imported canine case (from Spain) in 2006 [21]. According to the French reference center for leishmaniasis in 2017, an average of 180 cases of cutaneous leishmaniasis are reported in French Guiana each year: 85% due to *L*. *guyanensis* and 10% caused by *L*. *braziliensis* [19]. *Leishmania* species known so far in Guiana are *Leishmania guyanensis*, *L*. *braziliensis*, *L*. *amazonensis*, *L*. *naiffi* and *L*. *lainsoni* [21]. Another study conducted in French Guiana included 1017 new diagnosed cases of leishmaniasis records between 2006 and 2013 and showed 86.2% for *L*. *guyanensis*; 9.7% for *L*. *braziliensis*; 2.8% for *L*. *amazonensis*; and 1.3% for *L*. *lainsoni* [16]. Recently and for the first time in America, a case of human visceral leishmaniasis caused by *Leishmania siamensis*, acquired in Guyana, has been reported [37]. Another publication describes a clinical case in a cat from French Guiana with a nasal ulcer and ear nodules; molecular analysis identified *L*. *braziliensis* [38]. In our study, no *L*. *infantum* infection was detected in autochthonous French Guianan dogs sampled in 2008 and 2014, while 3.1% (3/98) were infected in 2016, all of them coming from Cayenne. It may suggest a recent emergence of this infection in this area. Dog is known to be the main reservoir of *L*. *infantum*, the causative agent of human and canine VL in South America [6]. The disease is also well known in the neighboring countries, such as Brazil, Suriname, Guyana, Venezuela, Colombia and *Lutzomyia longipalpis* is the main vector [39–41]. A study conducted in Northern Brazil, reports a prevalence of 13.1% in domestic dogs using IFAT [42].

Because French Guiana is a French territory and is part of the European Union, military dogs have come from metropolitan France (endemic territory for CanL) to Guyana for missions sometimes lasting a few months. During their stay in French Guiana, dogs can sometimes be involved in military missions taking place in the deep forest. As part of the follow-up of the canine vector-borne diseases, 78 MWD were enrolled between 2012 and 2018, before and after departure in French Guiana. *L*. *infantum* infection was detected in two MWDs, one in 2012 and one in 2016. In 2018, 2/16 were found positive and presented clinical signs of CanL. Parallel sequencing of the 18S rRNA, minicircle kDNA and ITS2 genes allowed the detection of *L*. *infantum* in these dogs. By contrast, no *Leishmania* infection has been reported in a serological survey on 119 dogs from French Guiana by IFAT conducted between 2006 and 2008 [43]. Consequently, the question of an emergence or of a newly recognized infection remains opened. Among infected dogs, only two MWDs presented clinical signs. CanL is a good example for the Iceberg phenomenon, since almost 50% of the affected canine population does not exhibit clinical signs [44]. Moreover, sick dogs manifest a variable and non-specific clinical spectrum [45], because CanL is a chronic and multisystemic disease that may potentially involve any organ [46]. In our study, apparently healthy infected dogs from Cayenne had a low or medium parasitemia (mean: 34 parasites/mL blood). By contrast, parasitemia in *Leishmania*-infected MWDs was low in two dogs and very high in two others, the higher parasitemia level being found in the sick dogs. It has been shown that dogs with medium to high parasitemia are sick or will eventually develop overt disease [47].

Bats are nocturnal and are the only mammals able to fly (sometimes seasonal migration), an important trait that can result in the dissemination of parasite species [48]. They are commonly infected by several trypanosomatid species, mainly from *Trypanosoma* genus: *T*. *cruzi*, *T*. *vespertilionis*, and *T*. *(Megatrypanum*) sp., among others [49] and by numerous *Leishmania* species [24]. It has been reported that bats, particularly numerous in French Guiana, are not reservoirs of *Leishmania* [50]. This may be due to the fact that French Guiana is a territory rich in large mammals, *Leishmania* parasites vectors are more attracted to these species than bats. In this study, 1.1% (1/92) of Guianan bats were infected. All blood samples from bats captured in 2013 were negative. *L*. *infantum* DNA was detected in a spleen of a short-tailed fruit bat *Carollia perspicillata*. To the best of our knowledge, there is only one report of the isolation of *Leishmania* (*L*. *infantum*) from the blood of this species, in Venezuela [51]. Two *Leishmania* species were identified in spleen and liver from Brazilian bats: *Molossus molossus* and *Glossophaga soricina* were found to be infected with *L*. *infantum* and *L*. *amazonensis*, and the latter was also found in *Molossus rufus*, *Nyctinomops laticaudatus*, *Eumops glaucinus*, *E*. *auripendulus*, *Artibeus literatus*, *Sturnira lilium* and *Myotis nigricans* [52]. *Leishmania (Viannia)* sp. was detected in a skin lesion from *G*.*soricina* and blood from *M*. *molossus* [53]. Recently, 6.4% (3/47) of Brazilian bats have been detected infected with *L*. *brazilinensis*, two *Platyrrhinus lineatus* and one *Artibeus planirostris* [54]. *Leishmania* spp. DNA was detected also in 8% of the 448 bat blood samples from a non-endemic region of leishmaniasis in Brazil, 41.6% of which were *L*. *infantum*, 38.9% *L*. *amazonensis* and 19.4% *L*. *braziliensis* [55]. Also, 9.8% (41/420) bats from Mexico were found infected by *L*. *mexicana* [56]. On the African continent, *Leishmania* kDNA was detected in 4.9% (8/163) of bats; *Leishmania* isolates from two bats were confirmed by ITS1 PCR to be *L*. *tropica* and *L*. *major*, isolated from two individual bats, *Cardioderma cor* and *Nycteris hispida*, respectively [57]. The low prevalence of *Leishmania* spp. infection in bats revealed in all these studies were in line with our investigation. By contrast, Shapiro *et al*. reported a 40% higher prevalence among bats from an endemic area of Brazil [54]. These results indicate that further studies are needed to assess the role of bats in maintaining the life cycle of leishmaniasis, especially in areas where these diseases are endemic.

The detection of the infection in autochthonous dogs, bats and in MWD in French Guiana suggests the possible existence of *L*. *infantum*-transmission cycle in this area. Many VL forms are asymptomatic in healthy humans, but symptomatic forms appear mainly in cases of malnutrition or the development of an immune deficiency (HIV in particular). Possible transmission cycle of *L*. *infantum* in French Guiana is therefore a threat for the Guyanese civil population, whose HIV prevalence is very high (1.0–1.5%) and is among the highest in the American continent [58]. Other investigations are required to fully understand the epidemiology.

### Conclusions

The detection of *L*. *infantum* in local dogs and bats from French Guiana, and in initially non-infected dogs coming from metropolitan France, suggests the possible existence of an autochthonous transmission cycle for visceral leishmaniasis. It highlights the need for active surveillance in domestic and wild animals, especially the potential reservoirs identified in this study and implementation of control measures. Competent vectors in this region remain to be identified.

## Supporting information

S1 Fig Phylogenetic tree constructed based on the sequences of 18S rRNA gene for isolates in this study and isolates of the Leishmania complex from GenBank database. Neighbor-joining tree was constructed from 18S rRNA partial gene using MEGA 7.0 software. The Kimura-2-parameter method was used. Numbers above branches correspond to bootstrap values based on 1,000 replicates. The analysis involved 37 nucleotide sequences. All positions containing gaps and missing data were eliminated. There were a total of 527 positions in the final dataset. Isolates were designated by their accession numbers in the beginning and their names.
(TIF)

## References

[1] Yamey G. Torreele E. The world's most neglected diseases. Brit Med J. 2002; 325(7357): 176–177. 10.1136/bmj.325.7357.176 12142292 PMC1123710

[2] Downing T, Votýpka J, Kuhls K, Lukeš J, Cannet A, Ravel C, et al *Leishmania* infections: Molecular targets and diagnosis. Mol Aspects Med. 2017; 1–29. 10.1016/J.MAM.2016.11.012 28159546

[3] Bern C, Maguire JH, Alvar J. Complexities of assessing the disease burden attributable to leishmaniasis. PLoSNegl Trop Dis. 2008; 2 10.1371/journal.pntd.0000313 18958165 PMC2569207

[4] Alvar J, Vélez ID, Bern C, Herrero M, Desjeux P, Cano J, et al Leishmaniasis worldwide and global estimates of its incidence. PLoS One. 2012; 7 10.1371/journal.pone.0035671 22693548 PMC3365071

[5] Kaszak I, Planellas M, Dworecka-Kaszak B. Canine leishmaniosis—an emerging disease. Ann Parasitol. 2015; 61:69–76. Available: http://www.ncbi.nlm.nih.gov/pubmed/26342500 26342500

[6] Dantas-Torres F. Canine leishmaniasis in South America. Parasit Vectors. 2009; (Suppl 1): S1 10.1186/1756-3305-2-S1-S1 19426440 PMC2679393

[7] Moreno J, Alvar J. Canine leishmaniasis: Epidemiological risk and the experimental model. Trends Parasitol. 2002; 18:399–405. 10.1016/S1471-4922(02)02347-4 12377257

[8] Alborzi A, Rasouli M, Shamsizadeh A. *Leishmania tropica*-isolated patient with visceral leishmaniasis in southern Iran. Am J Trop Med Hyg. 2006; 74:306–307. 74/2/306 [pii] 16474088

[9] Gállego M, Pratlong F, Fisa R, Riera C, Rioux JA, Dedet JP, Portús M. The life-cycle of *Leishmania infantum* MON-77 in the Priorat (Catalonia, Spain) involves humans, dogs and sandflies; also literature review of distribution and hosts of *L*. *infantum* zymodemes in the Old World. Trans R Soc Trop Med Hyg. 2001; 95(3):269–271. 10.1016/s0035-9203(01)90231-7 11490994

[10] Ready PD. Epidemiology of visceral leishmaniasis. Clin Epidemiol. 2014; 6:147–154. 10.2147/CLEP.S44267 24833919 PMC4014360

[11] Akhoundi M, Kuhls K, Cannet A, Votýpka J, Marty P, Delaunay P, et al A historical overview of the classification, evolution, and dispersion of *Leishmania* parasites and sandflies. PLoSNegl Trop Dis. 2016; 10:1–40. 10.1371/journal.pntd.0004349 26937644 PMC4777430

[12] Killick‐Kendrick R. Phlebotomine vectors of the leishmaniasis: a review. Med Vet Entomol. 1990; 4:1–24. 10.1111/j.1365-2915.1990.tb00255.x 2132963

[13] Floch H. *Leishmania tropica guyanensis* n. sp., agent de la leishmaniose tégumentaire des Guyanes et de l’Amérique Centrale. Ann Parasitol Hum Comp. 1954; 28:784–787. 14378914

[14] Carme B, Aznar C, Pradinaud R. Absence of a proven resurgence of Chagas disease or cutaneous leishmaniasis in French Guiana over the last two decades. Ann Trop Med Parasitol. 2001; 95:623–625. 10.1080/00034980120092561 11672468

[15] Dedet JP, Carme B, Desbois N, Bourdoiseau G, Lachaud L, Pratlong F. Épidémiologie des leishmanioses autochtones en France métropolitaine et d’outre-mer. Press Medicale. 2013; 42:1469–1481. 10.1016/j.lpm.2013.03.01016 23886932

[16] Simon S, Nacher M, Carme B, Basurko C, Roger A, Adenis A, et al Cutaneous leishmaniasis in French Guiana: Revising epidemiology with PCR-RFLP. Trop Med Health. Tropical Medicine and Health; 2017;45: 1–7. 10.1186/s41182-016-0041-6 28265182 PMC5331739

[17] Rotureau B. Ecology of the *Leishmania* species in the Guianan ecoregion complex. Am J Trop Med Hyg. 2006; 74:81–96. 74/1/81 [pii] 16407350

[18] Costa MM, Penido M, Santos MS, Doro D, de Freitas E, Michalick MSM, et al Improved canine and human visceral leishmaniasis immunodiagnosis using combinations of synthetic peptides in enzyme-linked immunosorbent assay. PLoSNegl Trop Dis. 2012; 6 10.1371/journal.pntd.0001622 22629475 PMC3358334

[19] Desjeux P. Information sur l’épidémiologie des leishmanioses et la lutte contre ces maladies par pays ou territoire, vol. 30 World Health Organization, Geneva, Switzerland 1991.

[20] Bastien P. Annual activity report of the national reference center for Leishmaniasis—Year 2017 (in French). Public Health France 2018; 72 p. https://cnr-leish.edu.umontpellier.fr/files/2018/09/Rapp_Act_2017_CNRL-et-annexes.pdf

[21] Rotureau B, Ravel C, Aznar C, Carme B, Dedet J. First report of *Leishmania infantum* in French Guiana: Canine visceral leishmaniasis imported from the Old World. J Clin Microbiol. 2006; 44: 1120–1122. 10.1128/JCM.44.3.1120-1122.2006 16517909 PMC1393124

[22] Baneth G, Aroch I. Canine leishmaniasis: A diagnostic and clinical challenge. Vet J. 2008;175: 14–15. 10.1016/j.tvjl.2006.11.011 17215150

[23] Jiménez M, González E, Iriso A, Marco E, Alegret A, Fúster F, et al Detection of *Leishmania infantum* and identification of blood meals in *Phlebotomus perniciosus* from a focus of human leishmaniasis in Madrid, Spain. Parasitol Res. 2013; 112: 2453–2459. 10.1007/s00436-013-3406-3 23535889

[24] Roque ALR, Jansen AM. Wild and synanthropic reservoirs of *Leishmania* species in the Americas. Int J Parasitol Parasites Wildl. 2014; 3: 251–262. 10.1016/j.ijppaw.2014.08.004 25426421 PMC4241529

[25] Figueiredo FB, Gremião IDF, Pereira SA, Fedulo LP, Menezes RC, Balthazar DA, et al First report of natural infection of a bush dog (*Speothos venaticus*) with *Leishmania* (*Leishmania*) *chagasi* in Brazil. Trans R Soc Trop Med Hyg. 2008; 102:200–201. 10.1016/j.trstmh.2007.10.001 18036627

[26] Luppi MM, Malta MCC, Silva TMA, Silva FL, Motta ROC, Miranda I, et al Visceral leishmaniasis in captive wild canids in Brazil. Vet Parasitol.2008; 155:146–151. 10.1016/j.vetpar.2008.04.024 18556130

[27] Paiz LM, Motoie G, Richini-Pereira VB, Langoni H, Menozzi BD, Tolezano JE, et al Antibodies and molecular detection of *Leishmania* (*Leishmania*) *infantum* in samples of free-ranging marmosets (Primates: *Callitrichidae*: *Callithrix* spp.) in an area of canine visceral leishmaniasis in South eastern Brazil. Vector Borne Zoonotic Dis. 2018 vbz.2018.2348 10.1089/vbz.2018.2348 30335584

[28] Otranto D, Cantacessi C, Pfeffer M, Dantas-Torres F, Brianti E, Deplazes P, et al The role of wild canids and felids in spreading parasites to dogs and cats in Europe Part I: Protozoa and tick-borne agents. Vet Parasitol. Elsevier B.V.; 2015;213: 12–23. 10.1016/j.vetpar.2015.04.022 26003669

[29] Lambson B, Smyth A, Barker DC. Leishmania donovani: Development and Characterisation of a Kinetoplast DNA Probe and Its Use in the Detection of Parasites 1. 2000;22: 15–22. 10.1006/expr.1999.4458 10631076

[30] Lachaud L, Marchergui-hammami S, Dereure J, Dedet JP, Lachaud L, Marchergui-hammami S, et al Comparison of Six PCR Methods Using Peripheral Blood for Detection of Canine Visceral Leishmaniasis Comparison of Six PCR Methods Using Peripheral Blood for Detection of Canine Visceral Leishmaniasis. 2002; 10.1128/JCM.40.1.210

[31] Mary C, Faraut F, Lascombe L, Dumon H. Quantification of *Leishmania infantum* DNA by a Real-Time PCR Assay with High Sensitivity. J Clin Microbiol. 2004;42: 5249–5255. 10.1128/JCM.42.11.5249-5255.2004 15528722 PMC525214

[32] Lachaud L, Chabbert E, Dubessay P, Dereure J, Lamothe J, Dedet JP, Bastien P. Value of two PCR methods for the diagnosis of canine visceral leishmaniasis and the detection of asymptomatic carriers. Parasitology. 2002; 125:197–207. 12358417 10.1017/s0031182002002081

[33] de Almeida ME, Steurer FJ, Koru O, Herwaldt BL, Pieniazek NJ, da Silva AJ. Identification of *Leishmania* spp. by molecular amplification and DNA sequencing analysis of a fragment of rRNA internal transcribed spacer 2. J Clin Microbiol. 2011; 49:3143–3149. 10.1128/JCM.01177-11 21752983 PMC3165567

[34] Kumar S, Stecher G, Tamura K. MEGA7: Molecular Evolutionary Genetics Analysis Version 7.0 for Bigger Datasets. 2016; 10.1093/molbev/msw054 27004904 PMC8210823

[35] World Organisation for Animal Health (OIE). Chapter 3.4.16. Manual of diagnostic tests and vaccines for terrestrial animals. 2018 http://www.oie.int/fr/normes/manuelterrestre/acces-en-ligne/

[36] Dedet JP. Cutaneous leishmaniasis in French Guiana. A review. Am J Trop Med Hyg. 1990; 43:25–28. 10.4269/ajtmh.1990.43.25 2200289

[37] Depaquit J, Kaltenbach ML, Gay F. Visceral leishmaniasis in traveler to Guyana caused by *Leishmania siamensis*, London, UK. Emerg Infect Dis. 2018; 24(8):1599–1600. 10.3201/eid2408.172147 30016253 PMC6056114

[38] Rougeron V, Catzeflis F, Hide M, De Meeus T, Bañuls AL. First clinical case of cutaneous leishmaniasis due to *Leishmania* (*Viannia*) *braziliensis* in a domestic cat from French Guiana. Vet Parasitol. 2011; 181:325–328. 10.1016/j.vetpar.2011.04.028 21570189

[39] Lainson R, Rangel BF. *Lutzomyia longipalpis* and the eco-epidemiology of American visceral leishmaniasis, with particular reference to Brazil—A review. Mem Inst Oswaldo Cruz. 2005; 100:811–827. 10.1590/s0074-02762005000800001 16444411

[40] Bauzer LGSR, Souza NA, Maingon RDC, Peixoto AA. *Lutzomyia longipalpis* in Brazil: A complex or a single species? A mini-review. Mem Inst Oswaldo Cruz. 2007; 102: 1–12. 10.1590/s0074-02762007000100001 17293992

[41] Travi BL, Tabares CJ, Cadena H, Ferro C, Osorio Y. Canine visceral leishmaniasis in Colombia: relationship between clinical and parasitological status and infectivity for sand flies. Am J Trop Med Hyg. 2001; 64(3–4):119–124. 10.4269/ajtmh.2001.64.119 11442205

[42] da Costa AP, Costa FB, Soares HS, Ramirez DG, de Carvalho Araújo A, da Silva Ferreira JIG, et al Environmental factors and ecosystems associated with canine visceral leishmaniasis in Northeastern Brazil. Vector-Borne Zoonotic Dis. 2015; 15:765–774. 10.1089/vbz.2015.1866 26684524

[43] de Broucker CA, Andréo V, Marié JL, Davoust B. Epidemiological surveillance of transmissible diseases on animals in the environment of soldiers in French Guyana. Int Rev Armed Forces Med Services. 2011; 84/2: 25–33.

[44] Ribeiro RR, Suzan M, Michalick M, da Silva ME, Peixoto CC, Santos D, et al Canine leishmaniasis: An overview of the current status and strategies for control. Biomed Res Int. 2018 10.1155/2018/3296893 29789784 PMC5896350

[45] Ribeiro RR, da Silva SM, Fulgêncio G de O, Michalick MSM, Frézard FJG. Relationship between clinical and pathological signs and severity of canine leishmaniasis. Rev Bras Parasitol Veterinária. 2013; 22:373–378. 10.1590/S1984-29612013000300009 24142168

[46] Solano-Gallego L, Miró G, Koutinas A, Cardoso L, Pennisi MG, Ferrer L, Bourdeau P, Oliva G, Baneth G, The LeishVet Group. LeishVet guidelines for the practical management of canine leishmaniosis. Parasit Vectors. 2011; 4:86 10.1186/1756-3305-4-86 21599936 PMC3125381

[47] Martínez V, Quilez J, Sanchez A, Roura X, Francino O, Altet L. Canine leishmaniasis: The key points for qPCR result interpretation. Parasit Vectors. 2011; 4:57 10.1186/1756-3305-4-57 21489253 PMC3086858

[48] Jones G, Teeling EC. The evolution of echolocation in bats. Trends Ecol Evol. 2006; 21:149–156. 10.1016/j.tree.2006.01.001 16701491

[49] Lima L, da Silva FM, Neves L, Attias M, Takata CSA, Campaner M, et al Evolutionary insights from bat trypanosomes: Morphological, developmental and phylogenetic evidence of a new species, *Trypanosoma* (*Schizotrypanum*) *erneyi* sp. nov., in African bats closely related to *Trypanosoma* (*Schizotrypanum*) *cruzi*and allied species. Protist. 2012; 163:856–872. 10.1016/j.protis.2011.12.003 22277804

[50] Rotureau B, Catzeflis F, Carme B. Absence of leishmania in Guianan bats. Am J Trop Med Hyg. 2006; 74:318–321. 74/2/318 [pii] 16474090

[51] de Lima H, Rodríguez N, Barrios MA, Ávila Á, Cañizales I, Gutiérrez S. Isolation and molecular identification of *Leishmania chagasi* from a bat (*Carollia perspicillata*) in northeastern Venezuela. Mem Inst Oswaldo Cruz. 2008; 103:412–414. 10.1590/s0074-02762008000400018 18661000

[52] Savani ESMM, de Almeida MF, de Oliveira Camargo MCG, d’Auria SRN, Silva MMS, de Oliveira ML, et al Detection of *Leishmania* (*Leishmania*) *amazonensis* and *Leishmania* (*Leishmania*) *infantum chagasi* in Brazilian bats. Vet Parasitol. 2010; 168:5–10. 10.1016/j.vetpar.2009.10.019 19939568

[53] Shapiro JT, da Costa Lima Junior MS, Dorval MEC, de Oliveira França A, Cepa Matos M de F, Bordignon MO. First record of *Leishmania braziliensis* presence detected in bats, Mato Grosso do Sul, southwest Brazil. Acta Trop. 2013; 128:171–174. 10.1016/j.actatropica.2013.07.004 23886850

[54] de Castro Ferreira E, Pereira AAS, Silveira M, Margonari C, Marcon GEB, de Oliveira França A, et al *Leishmania* (*V*.) *braziliensis* infecting bats from Pantanal wetland, Brazil: First records for *Platyrrhinus lineatus* and *Artibeus planirostris*. Acta Trop.2017; 172: 217–222. 10.1016/j.actatropica.2017.05.012 28502644

[55] Gómez-Hernández C, Bento EC, Rezende-Oliveira K, Nascentes GAN, Barbosa CG, Batista LR, Tiburcio MGS, Pedrosa AL, et al *Leishmania* infection in bats from a non-endemic region of Leishmaniasis in Brazil. Parasitology. 2017; 144(14):1980–1986. 10.1017/S0031182017001500 28831941

[56] Berzunza-Cruz M, Rodríguez-Moreno Á, Gutiérrez-Granados G, González-Salazar C, Stephens CR, Hidalgo-Mihart M, et al *Leishmania* (*L*.) *mexicana* infected bats in Mexico: Novel potential reservoirs. PLoSNegl Trop Dis. 2015; 9:1–15. 10.1371/journal.pntd.0003438 25629729 PMC4309399

[57] Kassahun A, Sadlova J, Benda P, Kostalova T, Warburg A, Hailu A, et al Natural infection of bats with *Leishmania* in Ethiopia. Acta Trop. 2015; 150:166–170. 10.1016/j.actatropica.2015.07.024 26232657

[58] Bello G, Nacher M, Divino F, Darcissac E, Mir D, Lacoste V. The HIV-1 subtype B epidemic in French Guiana and Suriname is driven by ongoing transmissions of pandemic and non-pandemic lineages. Front Microbiol. 2018; 9:1–12. 10.3389/fmicb.2018.00001 30108576 PMC6079251

