# Supplementary figures and images for: Potential animal reservoirs (dogs and bats) of human visceral leishmaniasis due to Leishmania infantum in French Guiana
Source: PLoS Negl Trop Dis. 2019 Jun 19;13(6):e0007456. doi: 10.1371/journal.pntd.0007456 (PMC6602241; doi:10.1371/journal.pntd.0007456)

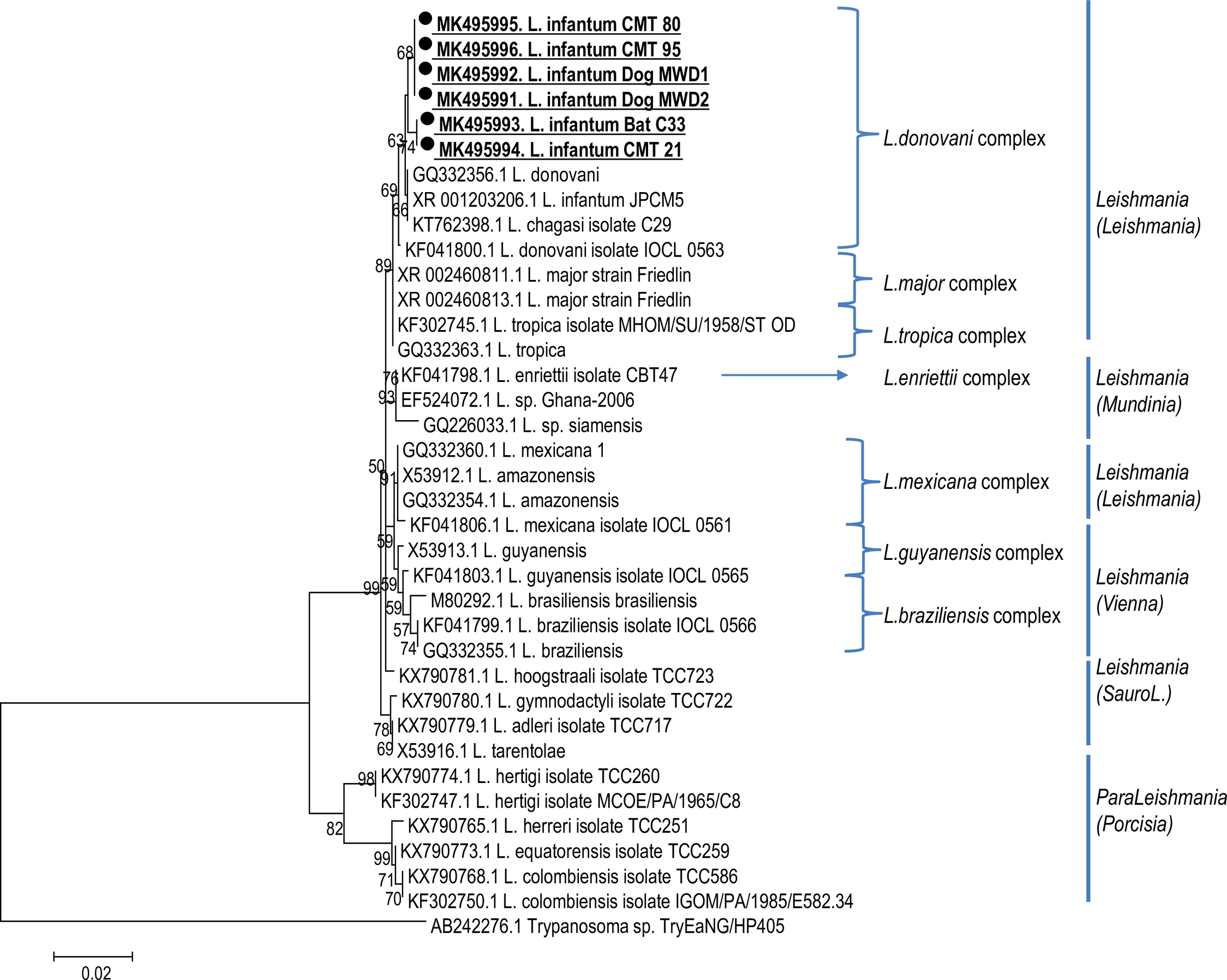

Supplement: S1 Fig — Neighbor-joining tree was constructed from 18S rRNA partial gene using MEGA 7.0 software. The Kimura-2-parameter method was used. Numbers above branches correspond to bootstrap values based on 1,000 replicates. The analysis involved 37 nucleotide sequences. All positions containing gaps and missing data were eliminated. There were a total of 527 positions in the final dataset. Isolates were designated by their accession numbers in the beginning and their names. (TIF) [file pntd.0007456.s001.tif]
